# Genome-wide analysis of yeast expression data based on a priori generated co-regulation cliques

**DOI:** 10.15698/mic2019.03.671

**Published:** 2019-01-22

**Authors:** Siyuan Sima, Lukas Schmauder, Klaus Richter

**Affiliations:** 1 Center for integrated protein research at the Department of Chemie, Technische Universität München, Lichtenbergstr. 4, 85748 Garching, Germany.

**Keywords:** coexpression, cliques, clusters, gene set enrichment, stress response, transcription factors, GO enrichment

## Abstract

DNA microarrays are highly sensitive tools to evaluate the gene expression status of organismic samples and standardized array formats exist for many different sample types. Differential expression studies usually utilize the strongest upor downregulated genes to generate networks visualizing the relationships among these genes. To include all yeast genes in one analysis and to get broader information on all cellular responses, we test *a priori* input of predefined genome-wide expression cliques and subsequent statistical analysis of the expression data. To this end, we generate a set of 72 co-regulation cliques using the information from 3196 microarray experiments. The obtained cliques performed highly significant in gene ontology and transcription factor enrichment analyses. We then tested the clique set on individual microarray experiments reporting on responses to pheromone, glycerol versus glucose based growth and the cellular response to heat. In all cases a highly significant determination of affected expression cliques was possible based on their average expression differences, the positions of their genes within hit rankings (UpRegScore) or the enrichment of the Top200 hits in certain cliques. The 72 cliques were finally used to compare experiments, which reported on the transcriptional response to polyglutamine proteins of different lengths. Using the predefined clique set it is possible to identify with high sensitivity and good significance sample and condition specific changes to gene expression. We thus conclude that an analysis, starting with these 72 preformed expression cliques, can complement traditional microarray analyses by visualizing the entire response on a static genome-wide gene set.

## INTRODUCTION

The single cell organism *Saccharomyces cerevisiae* is one of the best studied eukaryote model organisms [1] with a well annotated genome of approximately 5800 genes [2], of which 23% are homologous to humans [3]. It has more than 100 genes encoding regulatory proteins influencing directly the expression of other genes [4]. Combined with a short life cycle, *S. cerevisiae* therefore is widely used for biological network studies based on microarray data. Resulting networks can be based on protein-protein interactions (PPIs), signaling or metabolic connections or co-expression relationships [5-8]. They usually are visualized as nodes for genes, connected by edges, representing interactions between these nodes [9]. In most cases this approach leads to the grouping of connected nodes and the formation of coregulated cliques or clusters. Often co-regulated genes have been found to share also functional properties or even form protein complexes directly [10].

Several methods and organisms have been used to construct genomic or proteomic networks in recent years [3, 9, 11-19]. In some cases training sets of selected microarray experiments are used to derive connections between nodes and to make a network from them. In other cases databases, like STRING [20], COXPRESdb [21], modSEEK [22] or SPELL [23], are used, which contain information on co-regulation properties, but also may contain other types of interactions, like PPIs and co-naming in articles or abstracts [20]. After network construction, the identification of clusters, cliques or modules is the next important step. It is widely recognized that in most cases a change in the transcriptional program is not only affecting one or two clusters, but due to the connected nature of cellular responses it affects several transcriptional modules simultaneously and to variable extent. These clusters need to be well separated, especially if further analyses on isolated gene groups are planned, as in general enrichment analyses profits from high quality gene sets.

We previously had used genome-wide co-expression databases to generate networks with fairly high connection density from hits of microarray experiments. This approach was applied to the identification of differentially induced gene clusters after polyglutamine expression [24] or differentially expressed gene clusters after Hsp90-depletion in *C. elegans* [25]. These networks were constructed from the Top100 or Top200 hits per experiment and in all cases several co-regulation clusters could be separated from each other [24-26]. Nevertheless, in all cases some genes could not be connected within these networks even though they showed strongly altered expression behavior. Also, when trying to separate clusters within these networks, for some gene groups no significant gene ontology (GO)-term or transcription factor (TF) assignments could be obtained in enrichment analyses [24, 26]. This could be caused by the limitation to 100 or 200 Top-genes and the resulting exclusion of many important genes from these networks.

We here aim at analyzing the full genomic expression dataset. To do this, all yeast genes were first assigned to a number of co-regulated expression cliques by an unbiased classification algorithm. Thus, this network will now include all genes and even those that did not connect within the Top200-hit network in our previous experiments will be integrated [24, 26]. The resulting cliques then could prove useful to analyze microarray data in the context of the genome-wide response.

## RESULTS

### Highly significant yeast expression cliques can be derived from platform-specific coregulation data

To obtain genome-wide co-expression cliques for all yeast genes analyzed on GeneChip Yeast Genome 2.0 arrays we used publicly available information from previously reported experiments. We generated a co-regulation database based on the correlations within 3196 *S. cerevisiae* microarray experiments available for the GPL2529 platform in the GEO repository (Supplementary Figure S1). Using this database, termed ‘GPL2529full’, we generated a network, which contains 151,676 gene-gene connections in the full genome (Supplementary Figure S2) and then separated 72 expression cliques based on an optimized classification algorithm as described in the Material and Methods section (Supplementary Figure S3, Supplemental Table S1). The size of the cliques varied considerably with the smallest clique being six genes (SAN1-SKN7) and the largest clique being 775 genes (CUP5-TEF1). Most expression cliques were in the range of 15-100 genes and every yeast gene analyzed on the GPL2529 microarray platform was included exactly once.

**Figure 1:**
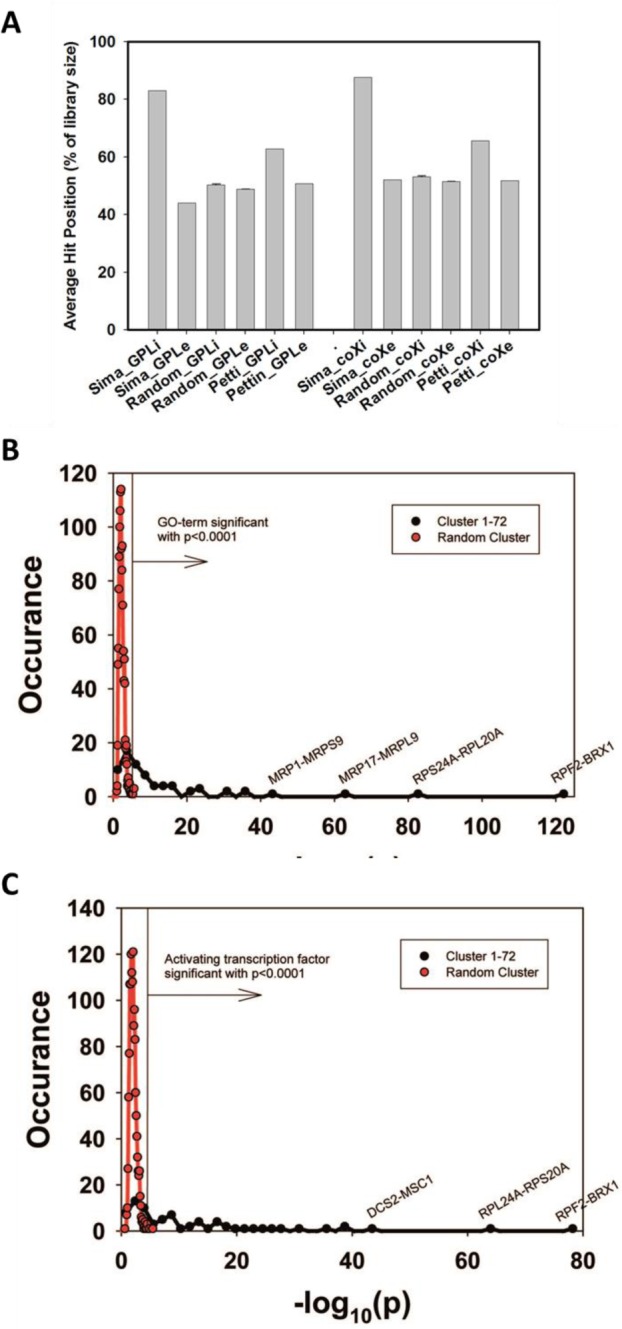
FIGURE 1: Evaluation of Genome-wide expression cliques. **(A)** Comparison between our clique set, random genes and the clique set from Petti *et al*. The comparison is based either on our own coexpression database (left side) or on the co-expression database from COXPRESdb (right side). **(B)** GO-term assignments for the 72 identified cliques or for random cliques. The significance threshold for p<0.0001 is indicated as vertical line in the figure. As comparison to the calculated cliques (red plot) random gene cliques were used (black plot). **(C)** Assignment of activating TFs for the identified 72 cliques or for random cliques based on the YEASTRACT database. The significance threshold for p<0.0001 is indicated as vertical line in the figure.

We first tested to what extent the isolated cliques indeed contain genes with high rankings in the co-expression database ‘GPL2529full’. Calculating the average intra-clique ranking and the average inter-clique ranking we find that the first value is much higher, showing that indeed the cliques accumulate preferentially co-expressed genes (**Figure 1A**). To exclude bias from using the same database for construction and evaluation of the cliques, we used another database, which is also publicly available (COXPRESdb) [21]. Here a similar spread is observable. In both cases this spread is eliminated, if randomly scrambled gene cliques are used (**Figure 1A**). Finally, we used this test to compare our clique set to another publicly available genome-wide clique set obtained with a different approach [29]. For this clique set the spread between intra- and inter-clique rankings is much smaller, suggesting that the clique set determined from our high-density networks could indeed show improved classification of the co-regulated genes.

We next performed analyses on the isolated cliques to evaluate functional correlations between the genes in each clique. To this end we first determined enriched GO-terms for each of the isolated gene groups (Supplemetary Table S2, the most prominent results are summarized in **Table 1**). Many of the determined p_Enriched_-values were in the (-log_10_)-range of +7 to +13, some even reaching a (-log_10_(p_Enriched_)) value of +124 (RPF2-BRX1, “nucleolus”). These values imply with very high confidence that the isolated co-expression cliques group genes of similar cellular functions. To evaluate the employed GO-term selection method, we tested scrambled expression cliques with the same enrichment evaluation method. For random cliques we observed mostly - log_10_(p_Enriched_) between +1 and +3 and even after 20 such scrambled clique-tests, the outstanding functional grouping of the genes in most co-expression cliques was obvious. We assumed that at p_Selection_<0.0001 (-log_10_(p_Selection_)>4), corresponding to a Z-score larger than 3.72, sufficiently high significance is achieved and this Z-score requires a -log_10_(p_Enriched_)>4.831 (**Figure 1B**, Supplemental Table S2). Many cliques in fact gave highly significant assignments, like the cluster RPS24A-RPL20A ((-log_10_(p_Enriched_)=+82, “cytoplasmic translation”) or the cluster MRP17-MRPL9 ((-log_10_(p_Enriched_)=+65, “mitochondrion organization”) or MRP1-MRPS9 ((-log_10_(p_Enriched_)=+42, “mitochondrion”). Many other cliques also were assigned with functions that obviously are correct (Supplemental Table S2). Altogether 4623 of the genes are included in cliques with very high significance (p<0.0001) and these are based on 1457 direct GO-term hits. The assignment of the most significant GO-terms to the separated gene cliques is available as supplementary table (Supplementary Table S2) with the respective p-values and the significance thresholds derived from the control experiments.

**TABLE 1.**
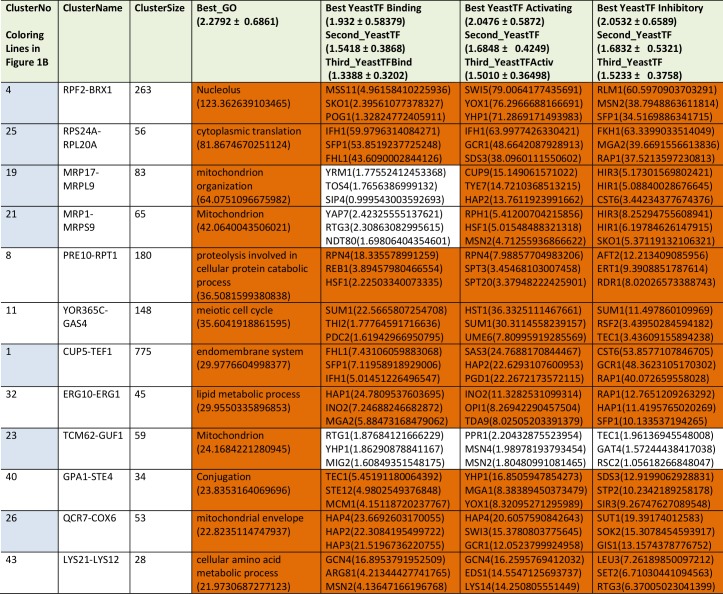
Most prominent results from GO-enrichment.

The cliques with p-values <10^-20^ are ranked according to their p-values. The data for all cliques are compiled in Supplemental Table S2.

We then performed a similar type of analysis to obtain information on potential TFs for the 72 expression cliques. We used the information from YEASTRACT to determine the three most likely TFs in the categories of “Binding”, “Activating” and “Inhibiting”. Here for most cliques the strongest enriched TF is supported by a (-log_10_(p_Enriched_)) in the range of +5 to +30 (Supplementary Table S2 most prominent in **Table2**, **Figure 1C**). When per-forming an analysis on 20 scrambled control separations, these values are considerably lower, implying that the applied separation method leads to gene cliques, whose regulation apparently can be linked to specific sets of TFs (**Figure 1C**). Based on average and standard deviation of the random sets, a Z-score of 3.72 is required and thereby our high significance threshold for the best TF requires a -log_10_(p_Enriched_)>4.103 (Supplemental Table S2). Best (-log_10_(p_Enriched_))-values in the category “activating TF” were obtained for the cluster RPF2-BRX1 (+79 for SWI5; +76 for YOX1 and +71 for YHP1) and RPS24A-RPL20A (+64 for IFH1; +48 for GCR1; +38 for SDS3). Altogether 4474 genes were included in cliques with at least one highly significant TF in the category of “activating TF”, with 1444 direct gene hits in the YEASTRACT database. Further TFs were assigned from the two other categories (all data in Supplemental Table S2). Thus the ability to assign common functional properties and specific TFs to most of the clustered genes suggests that the separation of the cliques correlates well with the transcriptional logic encoded in the yeast genome.

**TABLE 2.**
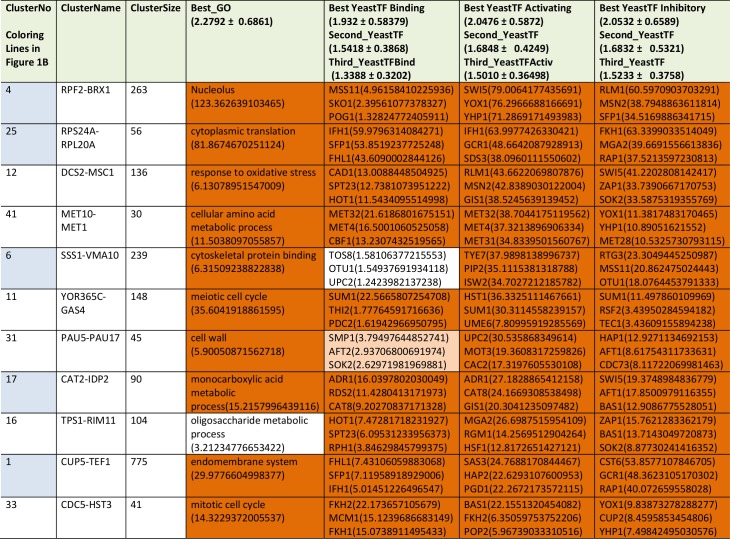
The most prominent results from TF-enrichment ranked according to their p-value in transcription factor activation.

Only cliques with p-values < 10^-20^ are included, the rest of the cliques are included in Supplemental Table S2.

### The *a priori* clusters provide detailed information on genome-wide transcriptional responses

We next aimed at testing microarray experiments, even if they were performed on other platforms (all information in Supplementary Table S3), to see whether the separation into these 72 cliques generally reflects the experimental realities in isolated experiments.

**Figure 2:**
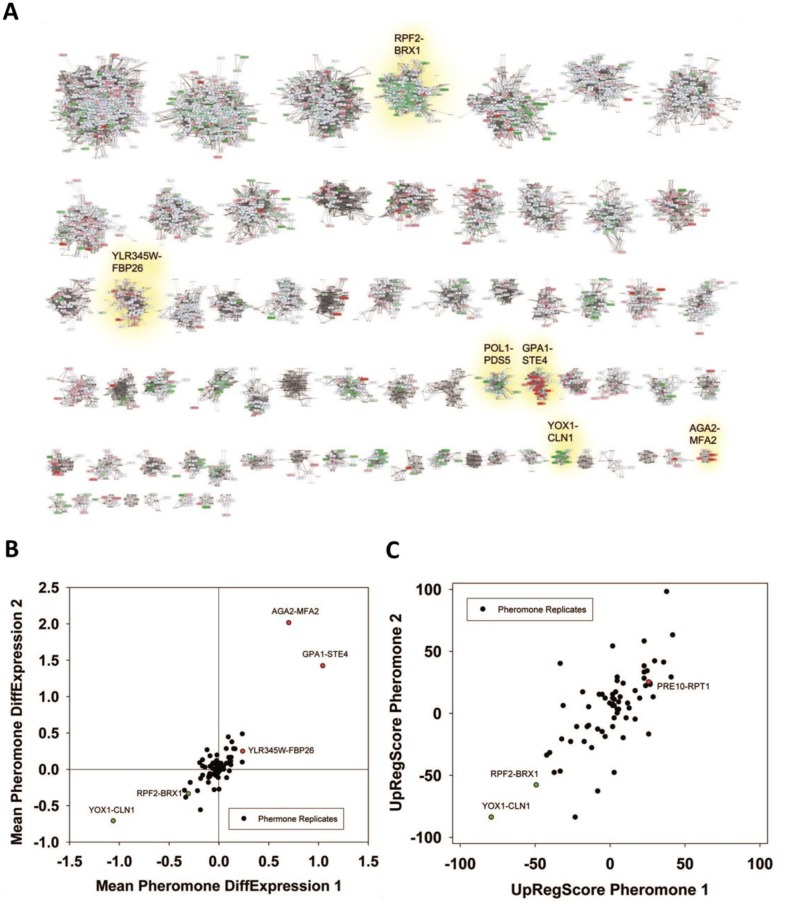
FIGURE 2: Analysis of microarray data sets on pheromone response. **(A)** Response to α-pheromone with data from [37]. Downregulation is shaded in four levels of green, upregulation in four levels of red. Genes, which are in the clustered network, but received no values in the experiment, were blanked out. Significantly changed cliques in replicate 1 are highlighted with yellow background and labelled with their clique name. **(B)** Comparison between two replicates in respect to average expression changes in the 72 cliques. We marked the cliques that are significantly changed in respect to average expression in both experiments (p<0.0001) in the plot with red (upregulation) and green (down-regulation) and labelled them accordingly. **(C)** UpRegScore for each cluster in the two replicates. Cliques, where both replicates showed significant shifts in the same direction were labelled in red (upregulation) or green (downregulation). We named them accordingly.

We first analyzed a reported response to α-pheromone (GSE7525, [37]). Plotting the expression difference of each gene onto the clustered network we find that indeed some of the cliques accumulate red and greenish colors (**Figure 2A**). We first used the Top200 hits in each direction to see, whether these preferentially fall into some of the 72 cliques.

This clearly is the case as judged from the enrichment factors and p-values derived from this analysis and in particular the clique GPA1-STE4 ((-log_10_(p)=+27) performs outstandingly, followed with a larger distance by the clique DCS2-MSC1 ((-log_10_(p)=+9). The cliques RPF2-BRX1 ((- log_10_(p)=+44), YOX1-CLN1 ((-log_10_(p)=+18) and SER1-ADE12 ((-log_10_(p)=+4.2) are significantly enriched for downregulated genes (Supplemental Table S4, **Table 3** with the most prominent results). To confirm the up- or downregulation of these cliques relative to the other gene cliques, we calculated the average expression differences and the UpRegScore. Based on random clique analysis we could obtain information on stochastic variations in these parameters and were able to derive significance parameters for them. We find the average expression differences in RPF2-BRX1 to be -0.30375 (probability for being not downregulated relative to random clusters: (-log_10_(p)=+23)) and upregulation in GPA1-STE4 (+1.046, (-log_10_(p)=+20)). YOX1-CLN1 is significantly downregulated (-1.0574, (-log_10_(p)=+15)). Several other cliques were also significantly shifted regarding their expression changes at lower, but still highly significant levels (-log_10_(p)>+4). We then compared the first with the second replicate. Here, we find that the similarity between these two experiments is very high and the correlation of expression differences (**Figure 2B**) and UpRegScores (**Figure 2C**) for the 72 cliques is almost linear. We marked the cliques that were significant in both experiments with the respective color, noting that especially small cliques are punished strongly due to their relative higher standard deviations (Supplementary Figure S4, S5). The strong correlation between the replicates shows that these two replicates yield very similar results in respect to the cliques induced or suppressed.

**TABLE 3.**
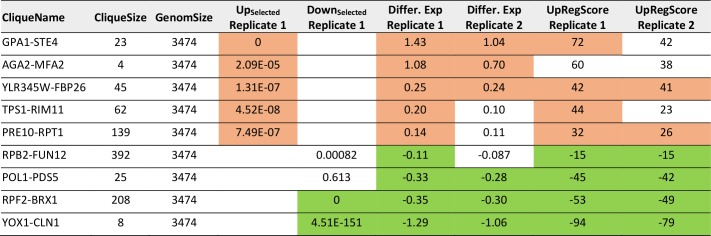
Most prominent hits from differential expression after pheromone induction within the 72 expression cliques for replicate 1 and replicate 2.

Results significantly above or below baseline (p<10^-4^) are colored in the categories for Top200-Enrichment of replicate 1, clique differential expression in both replicates or UpRegScore in both replicates.

**Figure 3:**
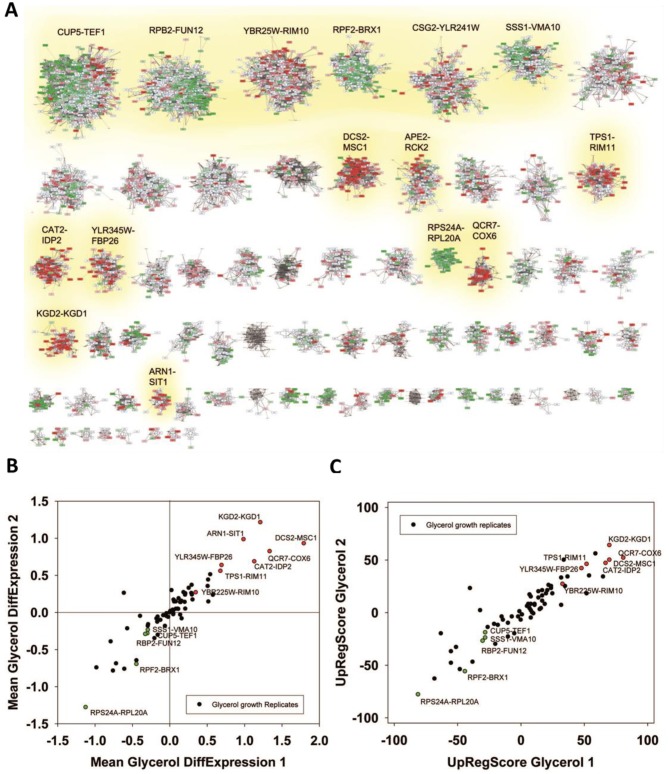
FIGURE 3: Analysis of microarray data sets on growth conditions. (**A**) Differences between glycerol-induced growth and glucose supported growth as described by [38]. Downregulation is indicated by four levels of green, upregulation by four levels of red. Significantly changed cliques of replicate 1 are highlighted with yellow background and labelled. (**B**) Correlation between two replicates and indication as to which values show significant upregulation in both replicates (red dots). The same is applied for downregulation (green labelling). (**C**) Comparison between two replicates regarding the UpRegScore of each clique. The labelling is performed as in Figure 2B.

We then performed the same analysis with a data set reporting on the differences between glycerol and glucose based growth (GSE6302, [38]). Visual inspection of the expression differences in the 72 cliques show that this response is producing much stronger expression changes than the response to pheromone and many more cliques appear systematically affected (**Figure 3A**). Here, as before, the identification of influenced cliques is possible based on their expression differences, the ranks of the genes in upregulation lists (UpRegScore) and the enrichment of the Top200 genes within the cliques (Supplemental Table S5, most prominent results in **Table 4**). Given that the response involves many more genes, the calculation of average expression differences for each clique appears very rewarding in addition to the Top200-enrichment. We find the cliques DCS1-MSC1 (+1.79, (-log_10_(p)=+139)) and QCR7-COX6 (+1.339, (-log_10_(p)=+27)) and CAT2-IDP2 (+1.133, (-log_10_(p)=+20)) to give the most significant upregulation and the cliques CUP5-TEF1 (-0.289, (-log_10_(p)=+18)), RPS24A-RPL20A (-1.12, (-log_10_(p)=+18)) and RPB2-FUN12 (- 0.32, (-log_10_(p)=+16)) to give the most significant downregulation response (**Figure 3B**). Also the UpRegScore yields highly significant p-values for each of those cliques (all (-log_10_(p)>+12)). We then tested, whether these cliques perform reproducibly in other biological replicates of this sample condition. Here, as before, the two replicates, which are available from the.pcl files on the SPELL server, strongly correlate in plots where the average expression differences (**Figure 3B**) or the UpRegScores (**Figure 3C**) of each clique are directly compared (Supplemental Table S5, most prominent results in **Table 4**).

**TABLE 4.**
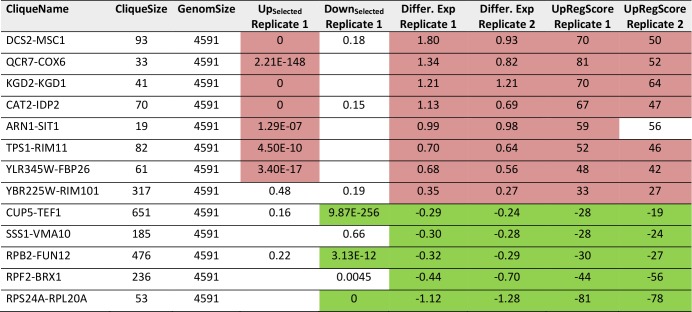
Most prominent hits from differential expression in glucose versus glycerol based growth within the 72 expression cliques for replicate 1 and replicate 2.

Results significantly above or below baseline (p<10^-4^) are colored in the categories for Top200-Enrichment of replicate 1, clique differential expression in both replicates or UpRegScore in both replicates.

**Figure 4:**
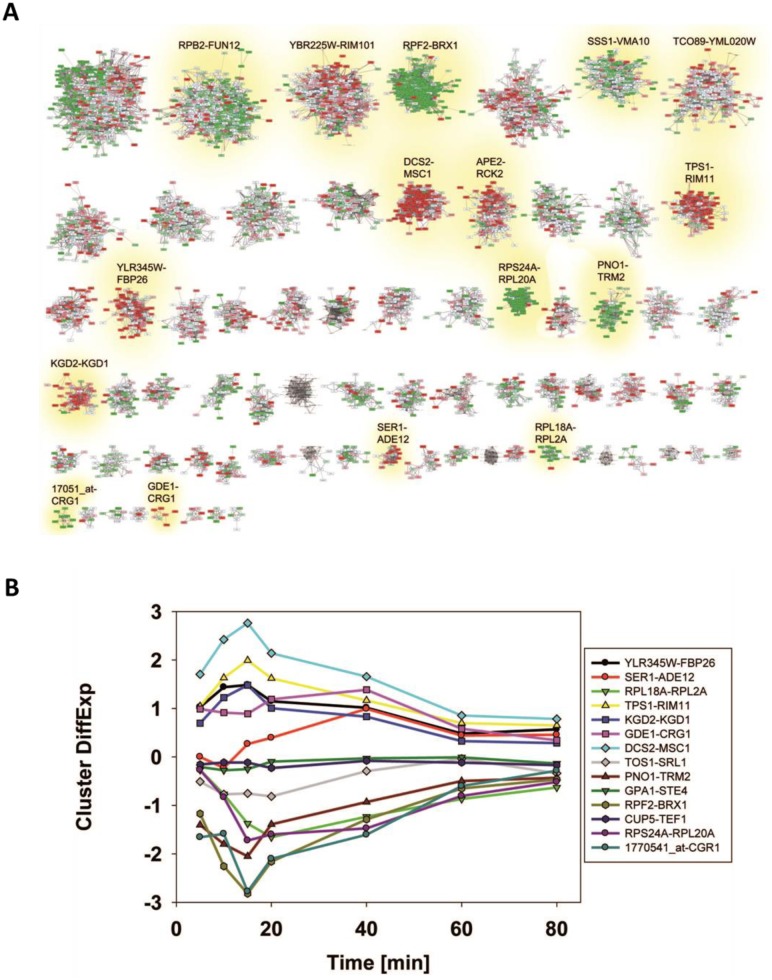
FIGURE 4: Analysis of microarray data sets on the heat-shock response. **(A)** Response to heat-shock after 40 minutes of recovery as reported by Gasch *et al*. [39]. Upregulation is indicated in four shadings of red. Downregulation is indicated in four shadings of green. Significantly changed cliques are highlighted with yellow background and labelled. **(B)** Time-course of the relevant cliques based on the single replicates provided on the SPELL webservice.

As a third test, we used the time series of the heat shock response as reported by Gasch *et al*. [39]. These data sets also were used previously by us to test our ability to connect the Top200 hits of the response [26], using the sample investigating the response 40 minutes after the heat-shock. In a previous analysis, here several hits were left outside of the connected network and their assignment to response parts was thus impossible from that network [26]. Using the same hits for enrichment analysis whether these preferentially fall into some of the 72 cliques. The selection of these cliques correlates well with the visual inspection of the response (**Figure 4A**, Supplemental Table S6 and **Table 5** for the most prominent results). The strongest enrichment of Top200 hits can be observed in DCS1-MSC1 (-log_10_(p)=+78), in TPS1-RIM11 (-log_10_(p)=+11.7) and in YLR345W-FBP26 (-log_10_(p)=6.5) next to the lower, but still significant shift in the cliques SER1-ADE1 (-log_10_(p)=5.9). Downregulation is observed most strikingly in RPF2-BRX1 (-log_10_(p)=+112), RPS24A-RPL20A (-log_10_(p)=+18), PNO1-TRM2 (-log_10_(p)=+7) and 1770541_at-CGR1 (-log_10_(p)=+6.5). These also represent the cliques with the most significant changes in average expression levels or UpRegScores (Supplemental TableS 6, most prominent results in **Table 5**). We used the average expression differences of interesting cliques to visualize the time course of the heat-induced response based on the single replicate arrays provided in the GEO repository. Here a clear pattern was observable, showing the very early induction of the DSC2-MSC1 and the TPS1-RIM11 clique that contains many genes of the classical heatshock response (**Figure 4B**). The clique RPF2-BRX1 containing nucleolus-related genes is already repressed five minutes after the heat-incubation, while the expression of ribosomal genes from RPL24A-RPL20A and RPL18A-RPL2A is reduced only after a short lag time. The significant upregulation of the SER1-ADE12 clique as observed 40 minutes after the heat-shock is characterized by an even longer lag time, implying that the heat-shock response actually is composed of waves of transcriptional changes affecting specific cliques with their own kinetics.

**TABLE 5.**
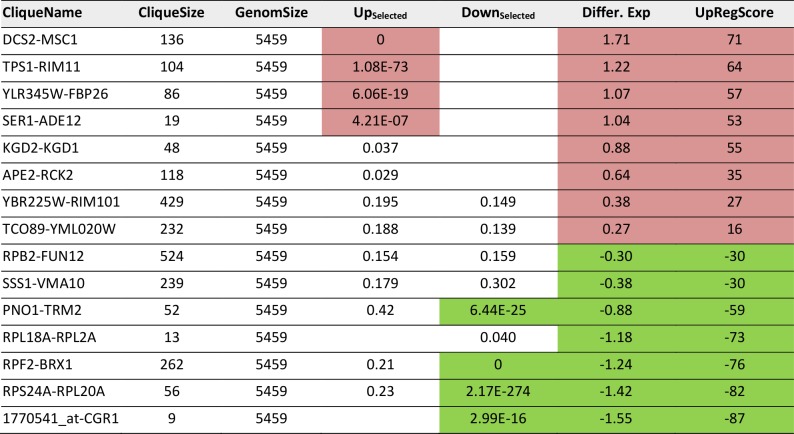
Most prominent hits from differential expression 40 minutes after heat shock within the 72 expression cliques.

Results significant above or below baseline (p<10^-4^) are colored in the categories for Top200-Enrichment, clique differential expression or UpRegScore.

The strong correlation between genes within one clique in these three experiments shows that many cliques generated by our connection and separation method are indeed regulated as transcriptional units. This confirms that the evaluation of individual microarrays with this type of clustered analysis nicely reflects the transcriptional response. Also it confirms that this analysis approach can help to investigate strong and weak responses alike based on p-values on all derived parameters.

### Full-genome analysis of the response to polyglutamine overexpression plasmids

**Figure 5:**
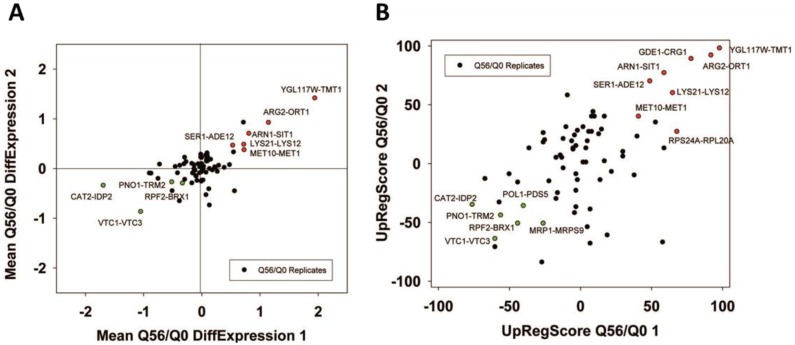
FIGURE 5: Analysis of the toxic effects of polyglutamine expression with pQ56. Correlation between two experiments of Q56-expression induced toxicity based on the average expression differences (A) or the UpRegScore (B) The clusters, which are significantly upor downregulated in both experiments, are highlighted in the respective color.

The 72 cliques present a good way to analyze the genomewide responses from expression data and they seem to work even in the treatment of single biological experiments. We thus felt that this analysis can extract relevant information from our own microarray samples. These arrays report on the overexpression of polyglutamine proteins of different length, with one of the constructs (Q56) producing a slow growth phenotype, while the other (Q30) does not induce growth defects. Experiments had been performed independently and maintained on the plates for 2-4 days before analysis to compensate for the different growth rates [24]. Employing the three analysis methods (Top200 Enrichment, Differential Expression, UpRegScore) on Q56/Q0, we find cliques with significant upor downregulations in the first experiment (Supplementary Table S7, most prominent in **Table 6**). These also match the visual inspection of the response (**Figure 5A**). In particular, these are CAT2-IDP2 (-1.69, -log_10_(p)=+96), DCS2-MSC1 (-0.72, -log_10_(p)=+35), QCR7-COX6 (-0.87, -log_10_(p)=+21), PNO1-TRM2 (-0.511, -log_10_(p) = + 19), YBR225W-RIM101 (-0.299, -log_10_(p)=+16), VTC1-VTC3 (-1.04, -log_10_(p)=+15) and others with weaker significance. In general, these cliques represent the large network, which had been assigned to the response to the nutritional status before [24], but now these genes are forming separate cliques. The upregulated genes include two large cliques with only mild upregulation: CUP5-TEF1 (0.271, - log_10_(p)=+30) and RPB2-FUN12 (0.186, -log_10_(p)=+20), which achieve significance based on their large size (775 genes and 524 genes) despite the small expression changes. There also are several smaller, but strongly affected cliques including YGL117W-TMT1 (1.94, -log_10_(p)=+16), LYS21-LYS12 (0.73, - log_10_(p)=+13), ARG2-ORT1 (1.15, -log_10_(p)=+13), ARN1-SIT1 (0.814, -log_10_(p)=+10) and MET10-MET1 (0.73, - log_10_(p)=+10). Most genes included in these cliques were identified before, but here likewise large parts of the response could not be assigned to GO-term or TF groups [24]. This is possible now that the hits are embedded into the context of their cliques. We compared the two experiments, which were incubated on agar plates for different times (Supplementary Figure S6A and B). Despite the different incubation, we still find a correlation for Q56, where the average expression differences of the cliques produce a roughly straight line (**Figure 5A** and **B**), hinting to consistent differences at least in the strongly affected cliques. So for the intoxicated sample, obviously the recorded response is consistent independently of the incubation time and sample condition.

**TABLE 6.**
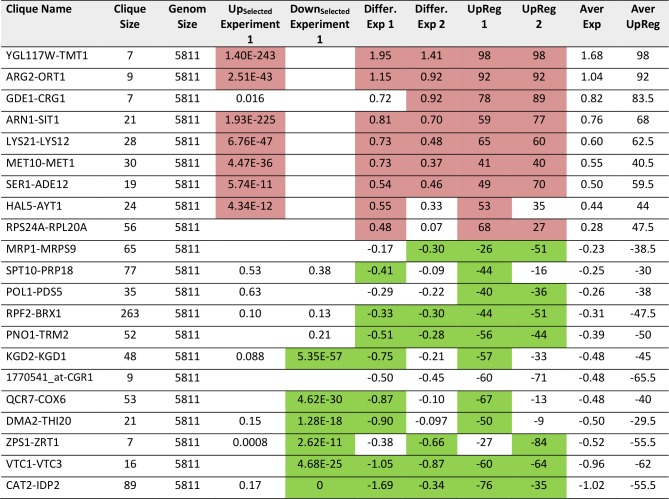
Most prominent hits from differential expression between Q56 and Q0 within the 72 expression cliques.

Results significant above or below baseline (p<10^-4^) are colored in the categories for Top200-Enrichment of experiment 1, clique differential expression in both experiments or UpRegScore in both experiments.

**Figure 6:**
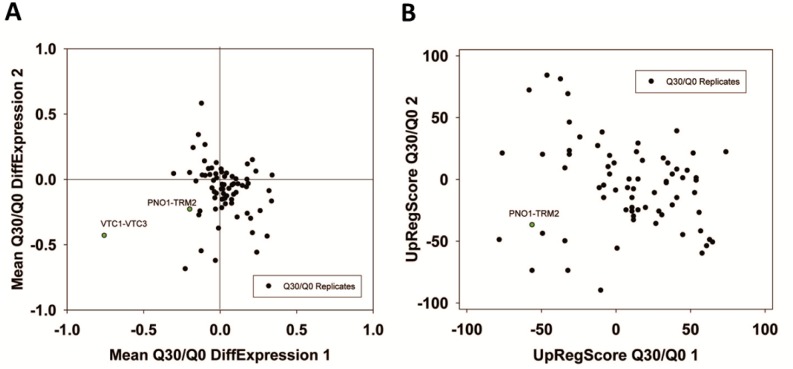
FIGURE 6: Analysis of non-toxic effects of polyglutamine expression with pQ30. (A) Correlation based on the expression differences and (B) the UpRegScore with the cliques being labelled, if significantly shifted in the same direction in both experiments.

For Q30, where no toxicity is observed, the expression differences are much smaller. This lead to difficulties when generating networks from the Top100-differentially expressed genes [24]. The visual inspection of the two experiments highlights that significant red or green cliques exist (Supplementary Figure S7A and B). Interestingly, while many cliques yield significant enrichments or expression shifts, only few of them show this behavior consistently in both experiments (Supplementary Table S8, most prominent cliques in **Table 7**). Clearly, no obvious correlation between the two experiments is observable (**Figure 6A** and **B**) and only few strongly altered cliques show consistent differential expression in both experiments. Nevertheless, each experiment shows its own significant difference between its Q30 and its Q0 control sample. We assume that due to the small influence of Q30 versus Q0 even small differences in growth conditions on the plates (e.g. different colony density) are masking the specific response. Apparently this effect can be stronger than the influence of the Q30-construct itself. Nevertheless, both experiments – based on the highly significant and visually observable shifts in some of their cliques - provide accurate information on the differences between the Q30 and Q0 samples in each experiment. Combining the analyses of both experiments, only the VTC1-VTC3 clique, the ZPS1-ZRT1 clique and the PNO1-TRM2 clique remain as candidates for a consistent influence from the overexpression of polyglutamine Q30. Interestingly, the PNO1-TRM2 clique is even downregulated in Q56 and Q30 samples alike. This had not been observed in the older analysis, possibly due to PNO1-TRM2 not having enough highly affected genes to generate a cluster of its own in the previously used methods [24]. Furthermore the weak upregulation of YGL117W-TMT1 and GDE1-CRG1 may be shared between Q56 and Q30-induced effects.

**TABLE 7.**
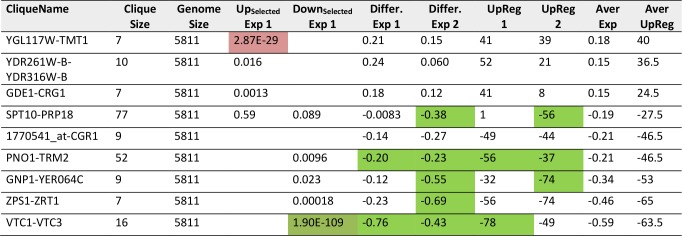
Most prominent hits from differential expression between Q30 and Q0 within the 72 expression cliques.

Results significant above or below baseline (p<10^-4^) are colored in the categories for Top200-Enrichment of experiment 1, clique differential expression in both experiments or UpRegScore in both experiments.

### GSEA yields gene sets with similarities to the strongest affected cliques

We finally aimed at testing whether other enrichment methods yield gene sets similar to the cliques identified here. To this end we used the same microarray experiments and evaluated them with the GSEA software. We included our cliques as additional gene sets in the gene set database and therefore obtained the enrichment scores also for them. For the response to α-pheromone we obtained several gene sets with high enrichment scores. Among them also our GPA1-SPE4 is present as second ranked and our clique YJL052C-A-YOR268C is ranked as number 20. When analyzing the 20 Top scoring gene sets, we find that eight from those sets are sharing at least 30% of their genes with GPA1-SPE4, implying that many of the identified gene sets are related to our clique GPA1-SPE4 (Supplemental Table S9). The same procedure was employed to compare Glycerol and Glucose based growth (Supplemental Table S10), for the heat-shock response (Supplemental Table S11) and for the experiments comparing either Q56 to Q0 (Supplemental Table S12) or Q30 to Q0 (Supplemental Table S13). In all cases many of the best gene sets from GSEA-analysis contain the genes also enriched in the relevant cliques from our clique set. In the other direction also our Top scoring cliques are represented directly by the Top scoring gene sets from GSEA. But the less strongly affected cliques from our clique set, which still show significant deviations from baseline, are usually not represented in the Top20 cliques of the GSEA-analysis. This implies that the clique set derived here may have advantages in uncovering significant but weakly responding sets due to its limitation to 72 static sets representing the full genome.

## DISCUSSION

### Application of the *a priori* clustered genome-wide co-expression cliques

We have used a genome-wide approach to analyze expression levels by generating co-expression cliques representing the full genome and then used the statistical performance of those in genome-wide expression analyses. Indeed, in every microarray experiment tested by us, the enrichment of upregulated and downregulated cliques is observable. This confirms that in all these experiments, even if expression changes are very low, like for the comparison between Q30 and Q0-expressing yeasts, we are still analyzing expression differences that are significantly above the noise threshold. The 72 co-expression cliques representing the full genome allow visualizing even very small shifts of individual cliques and thus provide significant information that may be lost otherwise. Also the analyses suggest that it may have advantages to analyze repeated experiments also individually without averaging the expression values prior to analysis as the individual analysis may better capture the influence of small differences between these repetitions. This level of detail could also not be achieved if genes are analyzed only as individual responders. Instead, in context of their clique, the concerted response of the clique enables significance tests on several levels and thus exposes even weak but concerted changes. Significance in our study has been achieved without the need to pre-filter the microarray data, without low signal probes being excluded, without manual readjustments to the cliques and without weighing factors on the probes. While we used defined parameter settings to produce the network (connections with the ten Top hits from the co-expression ranking) and to excise the cliques from it (protection threshold at five genes producing cliques of minimally six genes), an iterative optimization procedure could potentially improve the clique set quality further. It has to be realized though that producing smaller cliques will impact the ability to obtain significant results as the standard deviation increases strongly for smaller clique sizes (Supplementary Figure S4 and S5). Thus, we feel that the *a priori* clustered genome as presented here could be a good resource to perform a fast genome-wide analysis regarding the status of most of the important expression cliques encoded in the yeast genome. To enable general use of this analysis method, we made the network-files available and we included the analysis method into the webserver at www.clusterex.de.

Beyond the analysis of the expression data, the correlation of the 72 cliques with GO-terms and TFs interesting information on the molecular events that happen in the yeast cell. Several thousand genes could be directly related to the most prospective GO-terms or TFs for the 72 cliques. Potentially uncharacterized genes are assigned within this genome-wide clustering, placing them in cliques with well characterized genes and thereby providing a functional correlation at least in those cliques, where the GO-term assignment is very clear. These assignments are also potentially valuable to target the most prospective TFs: at least for the TFs IFH1 and GCR1, which are assigned to the clique RPL24A-RPS20A, there is indeed strong evidence that they are involved in regulation of ribosomal protein expression [40, 41].

In general, the main results compare well with other enrichment methods, like the gene-set-enrichment analysis GSEA. In comparison to this approach the 72 clique sets developed by us are static and represent the full genome, with one gene being assigned to one clique. In most cases tested here by us, the GSEA finds the gene sets with the highest enrichment and our top scoring cliques perform comparably. While GSEA is performed on more than 2000 gene sets, our cliques represent the entire genome within 72 cliques and make all its potentially observable responses accessible in a fast and efficient way. Even cliques with lower expression changes, which would show up only after a large number of gene sets when using the entire GSEA gene set database are readily observable here.

### Identification of cliques reporting on the presence of toxic and non-toxic polyglutamines

We finally used the group of co-expression cliques to visualize and re-evaluate the polyglutamine microarray experiments for which we had obtained expression values. pQ56-induced expression changes highlight strongly affected cliques, which contain many of the genes described before. Clearly significant in both experiments is the upregulation of the ARN1-SIT1 clique and the MET10-MET1 clique, which were previously identified as “iron-responsive” and “sulfur-responsive” [24]. The many genes not assigned to specific clusters in our study of the Top100 genes [24], now are enriched in the cliques ARG2-ORT1, YGL117W-TMT1, LYS21-LYS12 and SER1-ADE12. Also, we now can assign functions and potential TFs to the gene cliques that could not be assigned before [24]. The occurrence of those upregulated gene cliques in two different experiments (Experiment 1: Both samples three days on plate, Experiment 2: Q0 two days, Q56 four days on plates to compensate for the slow growth) implies that this result could be relatively stable over a broader range of incubation conditions. Similarly the downregulated cliques include the VTC1-VTC3 cluster previously assigned as “phosphor-related” [24], but also here the large number of genes, which previously were called “diauxic shift related” now can be assigned to the three clusters PNO1-TRM2, RPF2-BRX1 and CAT2-IDP2, separating these genes into nucleus, nucleolus and metabolism-related gene groups. In general, this analysis provides a more detailed description, even though this description does not include the fine structure of the genes within the cliques yet. From these results, we can conclude that several pathways are affected by the expression of the longer form of the polyglutamine constructs.

For the non-toxic Q30-YFP construct we also see significant expression differences in the two experiments. In general, these yeasts, which are not intoxicated, show much milder expression differences compared to Q56. Furthermore, only few clusters show reproducible responses in the two experiments. Nevertheless, each of the two experiments produces its own significant response. The strongest overlap between the experiments is the VTC1-VTC3 cluster, which is downregulated in both samples. None appears significantly upregulated in both experiments. This corresponds to our previous analysis based on the Top100 hits, where the VTC1-VTC3 cluster was identified, but no further significant changes could be extracted from the Top100 genes [24]. In the response of the cliques as presented here instead, additional cliques are significantly downregulated in both experiments: this is the ZPS1-ZRT1 clique and the PNO1-TRM2 cluster. Given that these cliques reacted twice in the Q56/Q0 experiments and twice in the Q30/Q0 experiments, it may well be systematically affected by the presence of polyglutamine proteins, but due to the very weak expression differences, this will require further experiments. Similarly, the clique GL117W-TMT1 and the clique GDE1-CRG1 are upregulated in both experiments and might require further reproduction due to their low expression changes.

Based on these examples, we expect that this analysis method is generally applicable for analyzing and comparing single experiments regarding their upor downregulated expression cliques on a genome-wide basis with an ability to detect weak, concerted and reproducible expression changes.

## MATERIALS AND METHODS

### Yeast co-expression database

To generate a co-regulation database specific for the GeneChip Yeast Genome 2.0 Array, we downloaded the 3493 GPL2529 datasets currently available in the GEO microarray repository, excluded the 297 experiments for *Schizosaccharomyces pombe*, and normalized the remaining ones with the software RMAExpress [27]. For each gene-gene pair the remaining 3196 value pairs were used to calculate the Pearson correlation coefficients by utilizing the ‘Correlation’ class of *science.dll* (www.sciencecode.net) [28]. The highest coefficients were indicative of the strongest expression correlation between two genes. These values were used to rank all coregulated partners for each yeast gene and store these rankings in a systematic database for 5813 Probe Sets. At that stage the database was translated to commonly used yeast gene names with the help of the information provided for the GPL2529 platform, retaining 5755 unique genes. Probe Set IDs were retained only, if no yeast gene name was assigned to the respective ID. This mostly was the case for Affymetrix control probes and the Probe Sets 1770455_at (ARG5,6), 1778252_at (ADE5,7), 1776844_at (PRM7), 1770541_at (SHL1) and 1778857_at (DUR1,2). As this newly generated database (termed ‘GPL2529full’) specifically uses information from GeneChip Yeast Genome 2.0 Arrays it contains data on all its 5755 individual genes.

### Clustering of all yeast genes

The co-expression database was used to generate a genomewide co-expression network containing all 5755 unique entries of the database with a procedure employed for limited gene numbers before [24-26] (Flow Diagram in Supplemental Figure S1). To this end we used the Top10 co-regulated genes from the database for each gene. This approach adds 351,055 connections to the network, of which 151,676 are different from each other. Despite the high number of edges and the average edge count of 2.31, the network density is only at 0.91% of the theoretical number of 16,557,135 connections between these 5755 yeast genes. Co-expression cliques were then identified and isolated from the network by a simple procedure: First, the whole gene-gene network matrix was sorted with the strongest gene-gene connection on the top and then treated according to four simple rules: 1) Starting from the top a new clique is created, if both genes are not included in a clique yet. 2) If one gene already is part of a previously defined clique, the second gene joins into this clique. 3) As long as cliques are fairly small (set to less than six genes), they are fused with larger ones, when they get connected via a new gene pair from the network matrix. 4) If both cliques are larger than five genes, instead, both genes remain as part of their previously assigned clique.

Each gene was uniquely assigned to a clique by this procedure. The cliques were then named according to the two genes with the highest number of intra-clique connections. In this procedure the clique number and composition depended mostly on two parameters: The gene-gene connections as set by the number of co-regulators used (here set to ten) and the clique separation as determined by the clique protection threshold (here set to larger than five). The employed clique separation method has been included for public use in the clusterex.de webserver. Furthermore the information on our genome-wide network and the clique set are available there. The assignment of genes to the respective cliques is also available as supplementary information to this manuscript in the form of a text file (Supplemental Table S1).

### Evaluation of the clique separation method

To evaluate the success of the clique separation we employed the ‘GPL2529full’ database. For each clique we calculated the average ranking in the co-expression lists for genes within the clique and towards genes from other cliques. The resulting values were compared to see, whether genes within the clique are indeed better positioned in the ranked lists of the database than genes from the other 71 cliques. This approach also was employed on randomly scrambled clique sets and on clique sets from other studies, in particular that from Petti *et al*. [29]. To prevent bias during this evaluation, which may arise from using the same database for network generation and for clique evaluation, we performed the same tests with the other publicly available database on yeast coexpression from COXPRESdb [21] (http://coxpresdb.jp/).

### Evaluation of isolated expression cliques

GO-enrichment and transcription factor (TF) enrichment were used to assess the quality of the clique separation and to assign the cliques to cellular functions. To this end each isolated gene clique was subjected to GO enrichment and TF enrichment analysis using the slim tables from http://geneontology.org/page/download-ontology [30, 31] and the flat tables from http://www.yeastract.com/formrankbytf.php [32, 33]. The enrichment calculation was done as described in https://github.com/ajmazurie/xstats.enrichment using the “hypergeometric_distribution” function. For each cluster the GO-term with the lowest p_Enriched_-value was recovered and for the TF enrichment the three TFs with the lowest p_Enriched_-values were retained in the disciplines of TF-binding, TF-activation and TF-inhibition. To obtain more information on the significance level of the selected GO-term or TF, the p_Enriched_-values were then compared to control experiments, which used same-sized cliques that contained randomly mixed genes. 20 such scrambled clique sets were usually analyzed to obtain average p_En-_ riched-values and standard deviations for the top-selected term or TF. These were used to estimate Z-scores and finally converted to p_Selection_-values that determine the significance of the employed term selection procedure. The Z-score to p_Selection_-value conversion was based on the implementation at https://github.com/HIPS/Probabilistic-Backpropagation/blob/master/c/PBP_net/pnorm.c.

### Genome-wide analysis of microarray samples

Experimental microarray data sets were obtained from our own experiments and from the SPELL-server [23] (https://spell.yeastgenome.org/). Expression values were exported to Cytoscape [34] to visualize the networks with the corresponding coloring of the genes. As such, we used thresholds of 0.25, 0.5, 0.75 and 1.0 to color the red-spectrum of the response and -0.25, -0.5, -0.75 and -1.0 to define the green spectrum. Average expression values and the UpRegScore as defined in Papsdorf *et al*. [26] were calculated for each clique. Furthermore, to compare also with previous analyses, the Top200 genes in each direction were used to determine their enrichment within the 72 cliques. This enrichment analysis was performed in similarity to the GO and TF-enrichment analyses and its results were described as (–log_10_(p)) for each clique. For the expression values and the UpRegScores we also performed 20 identical analyses from randomly scrambled clusters to estimate the significance of the deviation from baseline. The averages and standard deviations from random experiments were used to estimate Z-scores and p-values.

### Comparison to the GSEA method

The predefined genome-wide set of 72 cliques allows enrichment analyses in similarity to the commonly used gene set enrichment analysis (GSEA) [35] and the GlobalTest [36]. By including our cliques into the file containing all gene sets (Yeast_gene_set_Database_coexpression_gmt.gmt from http://ge-lab.org/gskb/), we compared the performance of our genome-wide clique set to the thousands of contributions from expression analyses already contained in this file. This modified database was used in GSEA analyses and the resulting enrichment scores (ES) of the large number of contributed gene sets were compared with the ES-scores of the cliques determined here. The relationship between the genes contained in the identified gene sets and our cliques was determined by testing, whether the top scoring gene sets contain the same genes as the top scoring cliques from the genome-wide clique set. To do this, we determine the five cliques that contain the highest number of genes from each identified gene set.

## SUPPLEMENTAL MATERIAL

All supplemental data for this article are available online at http://www.microbialcell.com/researcharticles/2019a-sima-microbial-cell/.

## Abbreviations:

GO: gene ontology,
TF: transcription factor.
